# Abnormalities of Mitochondrial Dynamics in the Failing Heart: Normalization Following Long-Term Therapy with Elamipretide

**DOI:** 10.1007/s10557-018-6805-y

**Published:** 2018-06-27

**Authors:** Hani N. Sabbah, Ramesh C. Gupta, Vinita Singh-Gupta, Kefei Zhang, David E. Lanfear

**Affiliations:** Department of Medicine, Division of Cardiovascular Medicine, Henry Ford Hospital, 2799 West Grand Boulevard, Detroit, MI 48202 USA

**Keywords:** Cardiolipin, Heart failure, Mitochondria, Myocardial energetics, Ventricular function

## Abstract

**Purpose:**

Abnormalities of MITO dynamics occur in HF and have been implicated in disease progression. This study describes the broad range abnormalities of mitochondrial (MITO) dynamics in Heart Failure with reduced ejection fraction (HF) and evaluates the effects of long-term therapy with elamipretide (ELAM), a MITO-targeting peptide, on these abnormalities.

**Methods:**

Studies were performed in left ventricular tissue from dogs and humans with HF, and were compared with tissue from healthy dogs and healthy donor human hearts. Dogs with HF were randomized to 3 months therapy with ELAM or vehicle. The following were evaluated in dog and human hearts: (1) regulators of MITO biogenesis, including endothelial nitric oxide synthase (eNOS), cyclic guanosine monophosphate (cGMP), and peroxisome proliferator-activated receptor gamma coactivator 1α (PGC-1α, a transcription factor that drives MITO biogenesis); (2) regulators of MITO fission and fusion, including fission-1, dynamin-related protein-1, mitofusion-2, dominant optic atrophy-1, and mitofilin; and (3) determinants of cardiolipin (CL) synthesis and remodeling, including CL synthase-1, tafazzin-1, and acyl-CoA:lysocardiolipin acyltransferase-1.

**Results:**

The study showed decreased levels of eNOS, cGMP, and PGC-1α in HF (dog and human). Increased levels of fission-associated proteins, decreased levels of fusion-associated proteins, decreased mitofilin, and abnormalities of CL synthesis and remodeling were also observed. In all instances, these maladaptations were normalized following long-term therapy with ELAM.

**Conclusions:**

Critical abnormalities of MITO dynamics occur in HF and are normalized following long-term therapy with ELAM. The findings provide support for the continued development of ELAM for the treatment of HF.

## Introduction

Mitochondria (MITO) are organelles with a well-known role in cellular metabolism and energy production, but they also play a critical role in cell growth and differentiation, cell signaling, and cell death or apoptosis [1, 2]. Abnormalities in MITO morphology/function have been observed in a variety of metabolic and cardiac diseases, including heart failure (HF). Once thought of as inert, mitochondria are now known to be highly dynamic, constantly undergoing biogenesis, fission and fusion in response to changes in energy demands [2, 3]. Fission and fusion are thought to be essential for normal mitochondrial function. A number of proteins and lipids have been shown to be important mediators of these dynamic processes [3], particularly peroxisome proliferator-activated receptor gamma coactivator 1α (PGC-1α), a transcription factor that drives MITO biogenesis, and cardiolipin (CL), a negatively charged phospholipid that is unique to MITO [2]. The failing heart shows to dysregulation in both fission and fusion regulating proteins, and downregulation of MITO fusion proteins enhances apoptosis; therefore, a possible contributor to ongoing cardiomyocyte loss [3] and potential mediator of progressive worsening of the HF state [4, 5]. Thus, agents that can normalize MITO fission and fusion may have important therapeutic potential in the treatment of HF.

Elamipretide (ELAM) (also referred to as SS-31, MTP-131, or Bendavia) is a water-soluble tetrapeptide with structural motifs of natural and synthetic amino acids [6]. The peptide enters the cell, crosses the MITO outer membrane, and localizes to the inner MITO membrane where it integrates with CL. In addition to modulating fission and fusion, CL plays an important role in the regulation of cristae formation, MITO DNA (mtDNA) stability and segregation, and the function and organization of the respiratory complexes into supercomplexes for oxidative phosphorylation [7–11] ELAM has been shown to enhance adenosine triphosphate (ATP) synthesis in multiple organs, including the heart, kidney, neurons, and skeletal muscle [12–21]. In dogs with coronary microembolization–induced HF, 3 months monotherapy with ELAM improved left ventricular (LV) systolic function and prevented progressive LV dilation. These improvements were associated with reduced reactive oxygen species (ROS) and overall improvement of MITO function that included improved MITO respiration, normalized membrane potential, and complex-I and -IV activities, as well as normalized rate of ATP synthesis [22]. The objective of the current study was to evaluate determinants of MITO dynamics, including biogenesis, fission/fusion, and CL synthesis and remodeling in HF, and the effect of long-term therapy with ELAM on these processes.

## Methods

### LV Tissue from Dogs with HF

Studies were performed using freshly frozen tissue samples obtained from the LV free wall of dogs with intracoronary microembolization–induced chronic HF as previously described [ 22, 23]. Briefly, 14 healthy mongrel dogs, weighing between 20.8 and 25.7 kg, underwent serial intracoronary microembolizations performed 1 to 2 weeks apart, to produce HF (LV ejection fraction ~30%) [23]. The study was approved by the Henry Ford Health System Institutional Animal Care and Use Committee and conforms to the *Guide for the Care and Use of Laboratory Animals* published by the US National Institutes of Health (NIH Publication No. 85–23, revised 1985). Six weeks after the last microembolizations, dogs with HF were randomized to 3 months of therapy with subcutaneous injections of ELAM (0.5 mg/kg once daily; *n* = 7; HF + ELAM) or to vehicle (normal saline administered once daily; n = 7); the latter group served as the control (HF-CON). LV tissue was harvested at the end of 3 months of therapy. LV tissue from 7 normal dogs was used in all studies for comparisons. All tissue samples from all study groups were obtained immediately upon removal of the heart and were rapidly frozen in isopentane pre-cooled in liquid nitrogen and stored at −70 °C until used. The effects of ELAM on hemodynamic, angiographic, echocardiographic, and plasma biomarker measures in HF dogs included in this study, have previously been described in detail [22].

### LV Tissue from Explanted Failed Human Hearts

Studies were also performed using freshly frozen tissue samples obtained from the LV free wall of 12 explanted failing human hearts. Among the 12 failing human hearts, 6 were failing due to ischemic etiology (ischemic cardiomyopathy [ICM]) and 6 were failing due to nonischemic etiology (idiopathic dilated cardiomyopathy [DCM]). Comparisons were made with LV tissue obtained from 6 normal donor (DNR) hearts deemed not suitable for transplantation. All tissue samples were obtained immediately upon organ explantation and were rapidly frozen in isopentane pre-cooled in liquid nitrogen and stored at −70 °C until used. All studies were approved by the Henry Ford Health System Institutional Review Board and all conformed with the principles outlined in the *Declaration of Helsinki*” (Br Med J 1964;2:177). All subjects gave written informed consent to participate in the study.

### Determinants of MITO Biogenesis

**Fig. 1:**
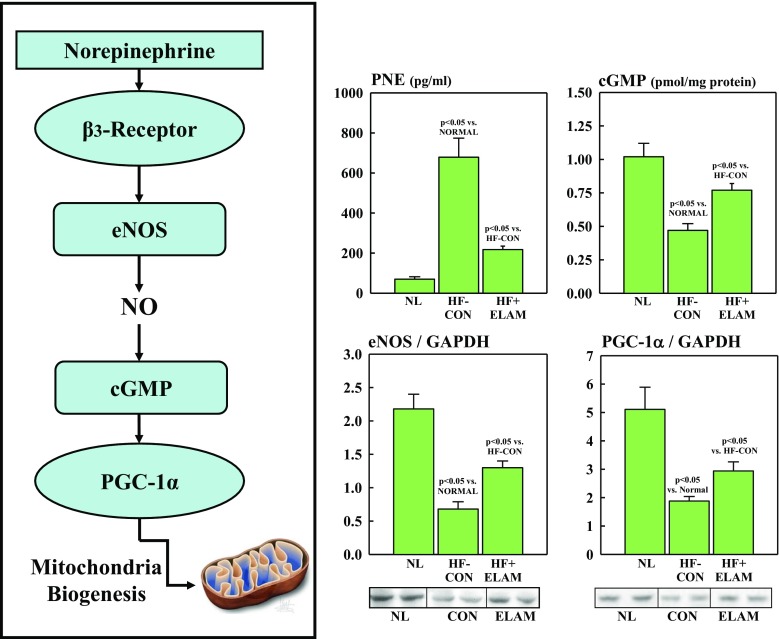
**Panel A**: Pathway whereby sympathetic drive mediates MITO biogenesis. **Panel B:** Dogs with HF had increased PNE and decreased levels of eNOS, NO, cGMP, and PGC-1α compared with NL. Treatment of HF dogs with ELAM (HF + ELAM) reversed these abnormalities. cGMP = cyclic guanosine monophosphate; du = densitometric units; ELAM = elamipretide; eNOS = endothelial nitric oxide synthase; HF = heart failure; HF-CON = dogs with HF who served as controls; HF + ELAM = dogs with HF who received treatment with ELAM; MITO = mitochondrial; NL = normal animals; NO = nitric oxide; PGC-1α = peroxisome proliferator-activated receptor gamma coactivator 1α; PNE = plasma norepinephrine

Peroxisome proliferator-activated receptor gamma coactivator 1α (PGC-1α) is a co-transcriptional regulation factor that induces MITO biogenesis by activating different transcription factors that drive transcription and replication of mtDNA. PGC-1α itself is regulated by upstream factors that include sympathetic activation. Enhanced and sustained activation of sympathetic drive triggers downregulation of endothelial nitric oxide synthase (eNOS), resulting in reduced elaboration of nitric oxide. This leads to a decrease in the activity of the second messenger cyclic guanosine monophosphate **(**cGMP**)** which, in turn, mediates downregulation of PGC-1α (Fig. 1). In the present study, eNOS and PGC-1α protein levels were measured in LV tissue homogenate from normal dogs, control dogs (i.e., HF-CON), and treated dogs (i.e., HF + ELAM) via Western blotting using specific antibodies (PGC-1α: Bethyl Laboratories, Inc., Montgomery, TX, USA, and eNOS: LifeSpan BioSciences, Inc., Seattle, WA, USA) and commercially available enzyme-linked immunosorbent assay (ELISA) kits. Bands were quantified in arbitrary densitometric units (du). Levels of cGMP in LV tissue homogenate from all 3 dog study groups were measured using an ELISA kit (Arbor Assays, Ann Arbor, MI, USA) and expressed in pmol/mg protein. Circulating levels of plasma norepinephrine (PNE) were quantified in plasma from all 3 dog study groups using a commercially available ELISA kit (MyBioSource, Inc., San Diego, CA, USA) and expressed in pg/ml.

### MITO Fission and Fusion Proteins

**Fig. 2:**
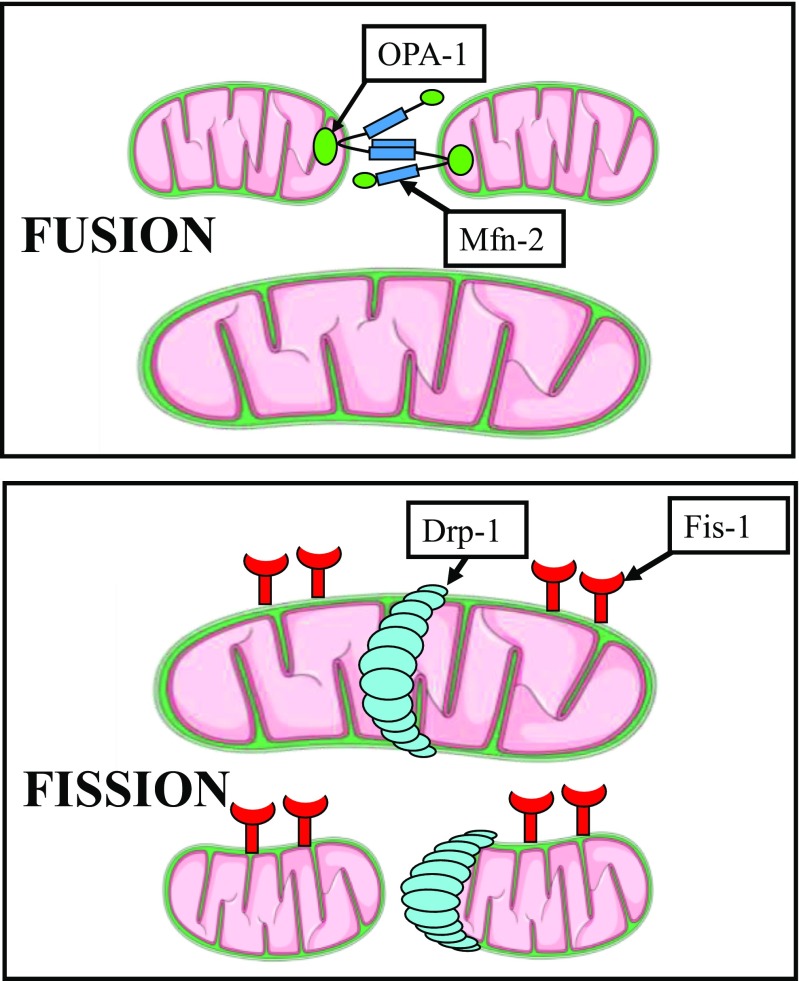
Fusion-mediating proteins include dominant OPA-1 and Mfn2 while fission-mediating proteins include Fis-1 and Drp-1. Drp-1 = dynamin-related protein-1; Fis-1 = fission-1; Mfn2 = mitofusin-2; OPA-1 = optic atrophyy-1

Levels of proteins that regulate MITO fission and fusion were measured in LV myocardium of all dogs and all explanted failed and DNR human hearts. Fission-mediating proteins included fission-1 (Fis-1) and dynamin-related protein-1 (Drp-1), and fusion-mediating proteins included mitofusin-2 (Mfn2) and dominant optic atrophy-1 (OPA-1) (Fig. 2). We also examined the phosphorylated form of Mfn2 (pMfn2), a regulator of fusion and mitophagy. In addition, we examined the regulation of a key regulatory protein mitofilin, a transmembrane protein of the inner MITO membrane that has critical functions in MITO morphology, MITO fission and fusion, and the formation of tubular cristae and cristae junctions. We also examined protein levels of glyceraldehyde 3-phosphate dehydrogenase (GAPDH) known to be unchanged in HF. In the present study, GAPDH was used as an internal control. All protein levels were measured in LV homogenate using Western blotting and bands quantified in densitometric units “du.” All protein measurements were normalized to GAPDH to adjust for loading conditions. Antibodies for Fis-1, Mfn2, and Drp-1 were purchased from Protein Biotechnologies Inc. (Tucson, AZ, USA) and for OPA-1 from Pierce Biotechnology, Inc. (Rockford, IL, USA). The antibody for pMfn2 was purchased from Millipore-Sigma (Burlington, MA, USA). Mitofilin antibodies were purchased from Novus Biologicals, LLC (Littleton, CO, USA).

### Determinants of CL Synthesis and Remodeling

We also examined abnormalities of determinants of CL synthesis and remodeling in the failing heart and the effects of therapy with ELAM on expression of these determinants. Total CL and (18:2)_4_CL species were measured using electrospray ionization mass spectroscopy [24] and were quantified in nmol/mg of non-collagen protein. Total CL and (18:2)_4_CL were normalized to LV MITO protein levels and were quantified as nmol CL/mg of MITO protein, as previously reported [22]. Protein level and messenger RNA (mRNA) expression of CL synthase-1 (CLS-1), an essential enzyme for the synthesis of CL, and the CL remodeling enzymes tafazzin-1 (TAZ-1) and acyl-CoA:lysocardiolipin acyltransferase-1 (ALCAT-1) were measured in LV tissue using Western blotting and reverse transcription-polymerase chain reaction. Western blotting bands were quantified in densitometric units (du) and mRNA expression in “fold change” from normal. All protein measurements were normalized to GAPDH to adjust for loading conditions. Antibodies for CLS-1, TAZ-1, and ALCAT-1 were obtained from abcam (Cambridge, MA, USA), Biorbyt, LLC (San Francisco, CA, USA), and Cell Signaling Technology, Inc. (Danvers, MA, USA), respectively.

### Statistical Analysis

Biochemical measures between normal, HF-CON, and HF + ELAM, as well as biochemical measures between DNR human hearts, human DCM hearts, and human ICM hearts, were compared using one way analysis of variance with alpha set at 0.05. If significance was attained, pairwise comparisons were performed using the Student-Newman-Keuls test, with *p* < 0.05 considered significant. All of the data exhibited normal distributions, and nonparametric testing led to similar results. Data are reported as mean ± standard error of the mean.

## Results

### MITO Dynamics in Explanted Failed Human Heart

**Fig. 3:**
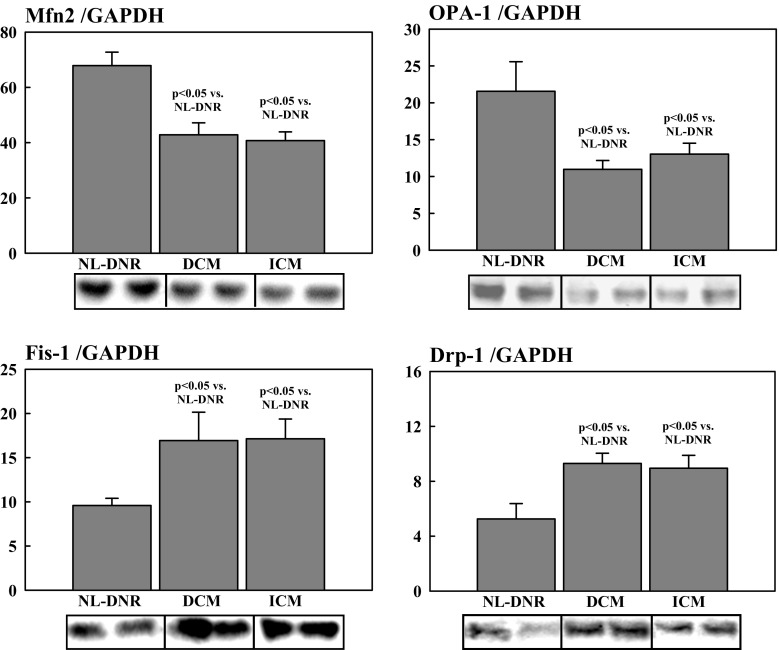
Levels of fusion-mediating proteins (i.e., OPA-1 and Mfn2) and fission-mediating proteins (i.e., Fis-1 and Drp-1) in DNR human hearts compared with human hearts with HF due to ICM and DCM. DCM = idiopathic dilated cardiomyopathy; DNR = normal donor; Drp-1 = dynamin-related protein-1; du = densitometric units; Fis-1 = fission-1; HF = heart failure; ICM = ischemic cardiomyopathy; Mfn2 = mitofusin-2; NL = normal animals; OPA-1 = optic atrophyy-1

**Fig. 4:**
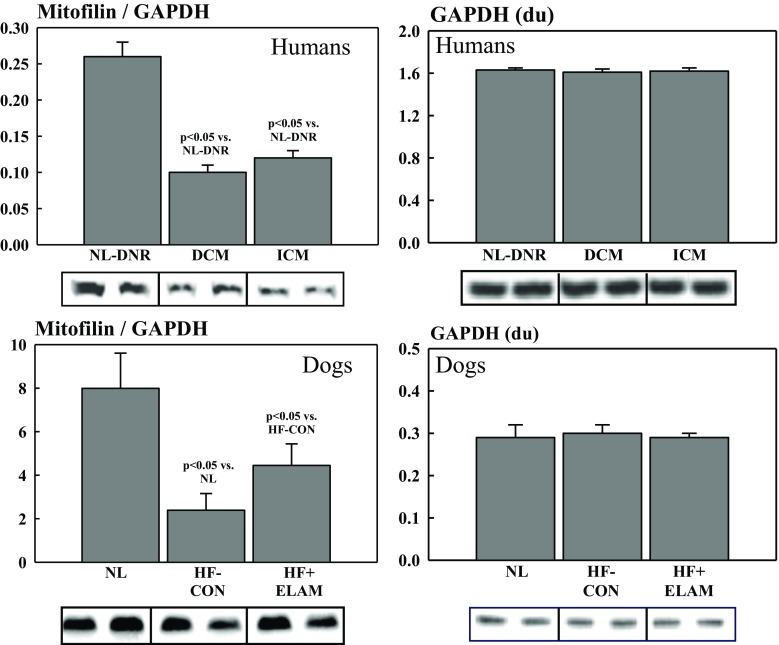
Levels of the regulatory protein mitofilin and the control protein GAPDH in DNR human hearts compared with human hearts with HF due to ICM and DCM (**top panels**) and in NL dogs, dogs with HF (HF-CON), and dogs with HF treated with ELAM (HF + ELAM; **bottom panels**). DCM = idiopathic dilated cardiomyopathy; DNR = normal donor; du = densitometric units; ELAM = elamipretide; GAPDH = glyceraldehyde 3-phosphate dehydrogenase; HF = heart failure; HF-CON = dogs with HF who served as controls; HF + ELAM = dogs with HF who received treatment with ELAM; ICM = ischemic cardiomyopathy; NL = normal animals

Studies of measures of MITO dynamics in tissue from explanted failed human hearts were conducted to ensure that the abnormalities seen in human tissue were consistent with those seen in dogs with experimentally induced HF. All patients were on optimal standard of care therapy for HF with reduced ejection fraction up until the date of cardiac transplantation, and none were receiving or had received ELAM in any form. Compared with DNR hearts, the level of PGC-1α protein was significantly lower in hearts of DCM (0.28 ± 0.05 vs. 0.91 ± 0.10; *p* < 0.05) and ICM (0.17 ± 0.03 vs. 0.91 ± 0.10; p < 0.05) etiology. There was no significant difference in the extent of downregulation of PGC-1α between DCM and ICM aetiologies’. There was a marked degree of dysregulation with respect to proteins directly involved in the dynamics of MITO fission and fusion. Among fission-regulating proteins, Fis-1 and Drp-1 were significantly increased in DCM and ICM hearts compared with DNR hearts (Fig. 3). In contrast, among fusion-regulating proteins, Mfn2 and OPA-1 were significantly reduced in DCM and ICM hearts compared with DNR hearts (Fig. 3). There were no statistical differences between DCM and ICM hearts with respect to any of the fission or fusion proteins that were examined (Fig. 3). Levels of the inner membrane protein mitofilin were also significantly reduced in DCM and ICM hearts compared with DNR hearts (Fig. 4). Again, there were no statistical differences between DCM and ICM hearts with respect to levels of mitofilin in the LV myocardium. The internal control protein GAPDH was essentially unchanged in DCM and ICM hearts compared with DNR hearts *(*Fig. 4).

### MITO Dynamics in LV Myocardium of Dogs with HF

Compared with normal dogs, HF-CON dogs manifested a marked and significant increase in PNE indicative of enhanced sympathetic activation (Fig. 1). The increase in PNE was accompanied by a significant reduction in eNOS and cGMP in LV myocardium of HF-CON dogs compared with normal dogs (Fig. 3). These alterations of signaling molecules gave rise to downregulation of PGC-1α protein in LV myocardium of HF-CON dogs compared with normal dogs (Fig. 3).

**Fig. 5:**
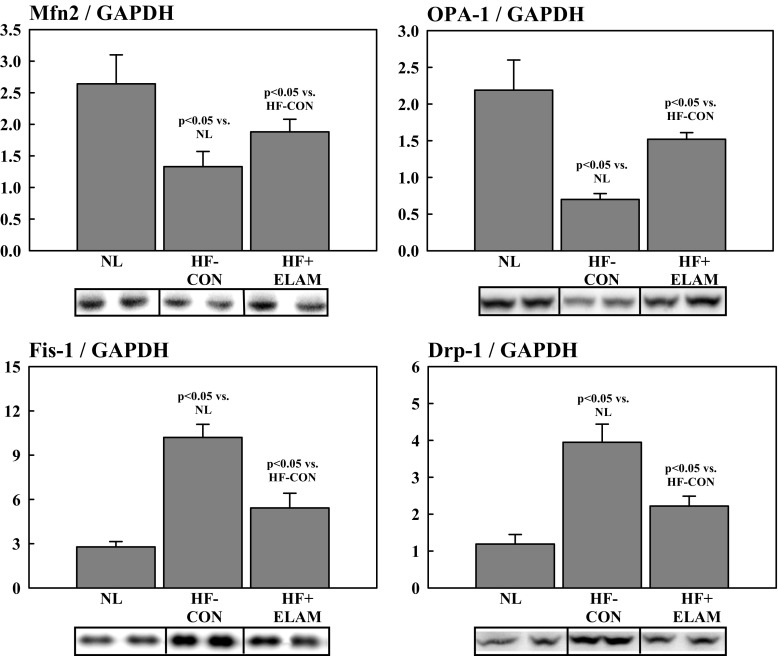
Levels of fusion-mediating proteins (i.e., OPA-1 and Mfn2) and fission-mediating proteins (i.e., Fis-1 and Drp-1) in NL dogs, dogs with HF (HF-CON), and dogs with HF treated with ELAM (HF + ELAM). Drp-1 = dynamin-related protein-1; du = densitometric units; Fis-1 = fission-1; HF = heart failure; HF-CON = dogs with HF who served as controls; HF + ELAM = dogs with HF who received treatment with ELAM; Mfn2 = mitofusin-2; NL = normal animals; OPA-1 = optic atrophyy-1

Compared with normal dogs, dogs with untreated HF who served as the controls showed a marked degree of dysregulation of proteins directly involved in the dynamics of MITO fission and fusion. As with failing human hearts, failing dog hearts showed a marked and significant increase in fission-regulating proteins Fis-1 and Drp-1 compared with normal hearts (Fig. 5). In contrast, and also consistent with observation in failing explanted human hearts, the levels of fusion-regulating proteins Mfn2 and OPA-1 were markedly and significantly reduced in DCM and ICM hearts compared with normal hearts (Fig. 5). Levels of the inner membrane protein mitofilin were significantly reduced in HF-CON dogs compared with normal dogs (Fig. 4). As with human tissue, the internal control protein GAPDH was essentially unchanged in HF-CON compared with normal LV dog tissue (Fig. 4).

### Effects of ELAM on MITO Dynamics in LV Myocardium of Dogs with HF

We previously showed that long-term (3 months) treatment with ELAM in dogs with HF improved LVEF and chamber remodeling and normalizes MITO function, complex activity, and maximum rate of ATP synthesis, while normalizing the rate of formation of ROS [22]. In the present study, long-term therapy with ELAM in dogs with HF (HF + ELAM) significantly reduced PNE concentration and significantly increased protein levels of cGMP, eNOS, and PGC-1α compared with untreated HF-CON dogs (Fig. 1). Compared with HF-CON dogs, HF + ELAM dogs showed significantly reduced protein levels of Fis-1 and Drp-1 and significantly increased protein levels of Mfn2 and OPA-1 (Fig. 5). Compared to normal dogs, pMfn2 was significantly decreased in HF-CON (1.01 ± 0.04 vs. 4.31 ± 0.65, *p* < 0.05). Treatment with ELAM tended to restore pMfn2 to near normal levels (2.24 ± 0.34), albeit partly. When normalized to total Mfn2 protein, pMfn2 in HF-CON was again significantly lower than in normal dogs (2.91 ± 0.86 vs. 6.27 ± 0.86, p < 0.05) and was restored to near normal level in HF-ELAM dogs (4.22 ± 0.54). Levels of the inner membrane protein mitofilin were also significantly increased in HF + ELAM dogs compared with HF-CON dogs (Fig. 4). In contrast, the internal control protein GAPDH was essentially unchanged in HF + ELAM compared with HF-CON LV dog tissue (Fig. 4).

### Abnormalities of CL in Dogs with HF and Effects of Therapy with ELAM

**Fig. 6:**
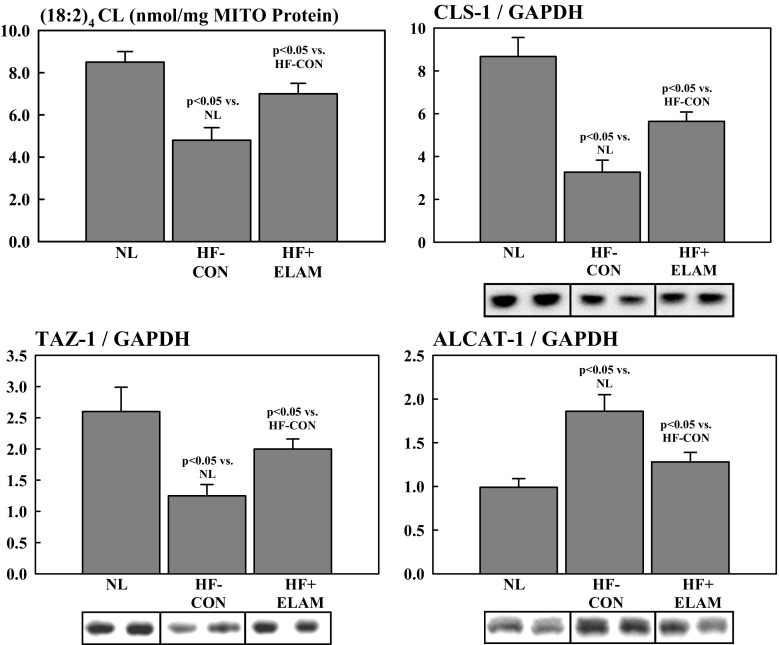
Levels of CL species ([18:2]_2_ CL), CLS-1, and the CL remodeling enzymes TAZ-1 and ALCAT-1 in NL dogs, dogs with HF (HF-CON), and dogs with HF treated with ELAM (HF + ELAM). ALCAT-1 = acyl-CoA:lysocardiolipin acyltransferase-1; CL = cardiolipin; CLS-1 = cardiolipin synthase-1; du = densitometric units; ELAM = elamipretide; HF = heart failure; HF-CON = dogs with HF who served as controls; HF + ELAM = dogs with HF who received treatment with ELAM; MITO = mitochondrial; NL = normal animals; TAZ-1 = tafazzin-1

As alluded to earlier, CL is a key regulator of MITO biogenesis and fission and fusion dynamics, and is also the target for ELAM therapy in HF. We previously showed that long-term therapy with ELAM normalizes total levels of CL and levels of (18:2)_4_CL in dogs with chronic HF (Fig. 6) [22]. In the present study, CLS-1, a key enzyme in the synthesis of CL, was significantly reduced in HF-CON dogs compared with normal dogs and was normalized after therapy with ELAM (Fig. 6). We also showed that TAZ-1 and ALCAT-1, important CL + remodeling enzymes, are dysregulated in HF. Specifically, TAZ-1 was significantly reduced and ALCAT-1 was significantly increased in HF-CON dogs compared with normal dogs and that long-term treatment with ELAM normalized protein levels of both enzymes (Fig. 6).

## Discussion

Results of this study indicate that in dogs and humans with HF, the LV myocardium manifests impaired MITO dynamics evidenced by impaired MITO biogenesis, dysregulation of the MITO fission and fusion machinery, and downregulation of mitofilin, a key protein necessary for the formation of tubular cristae and cristae junctions. In patients with HF, these abnormalities are present regardless of the etiology of HF, namely ICM or DCM. Importantly, results of this study showed that these defects can be reversed/normalized by long-term therapy with ELAM, a first-in-class cell-permeable tetrapeptide that selectively targets mitochondria. From a mechanism of action viewpoint, ELAM is known to integrate with and bind to CL, a constituent of the MITO inner membrane and site of the ETC.

In dysfunctional mitochondria typical of diseases such as HF, stability and integrity of CL are essential given the central role of CL in the regulation of MITO cristae formation, MITO fission and fusion, mtDNA stability and segregation, and in the function and organization of the respiratory complexes into supercomplexes for oxidative phosphorylation [7–11]. Preserving the integrity of CL at sites of curvature along the MITO inner membrane serves to preserve constituent complex proteins of the ETC that are essential for efficient electron transfer along the ETC that led to normalization of energy production by the MITO and prevention of excess generation of ROS also by the mitochondria [15, 20]. Efficient oxidative phosphorylation is also dependent on reducing ROS production by mitochondria. Excess formation of ROS is the primary culprit in the disruption of CL leading to MITO dysfunction. The (18:2)_4_ acyl chain configuration of CL with the 18-carbon fatty alkyl chains with 2 unsaturated bonds on each are primary targets for ROS. Studies in dogs with chronic HF showed that long-term therapy with daily SC injections of ELAM can improve LV systolic function, a desirable outcome that was accompanied by normalization of plasma biomarkers and by improved MITO function evidenced by normalization of MITO respiration, membrane potential, complex-I and -IV activities, ROS formation, and maximum rate of ATP synthesis and ATP/adenosine diphosphate ratio [22]. The current study builds on these findings, showing that ELAM in the setting of HF also normalizes MITO dynamics as evidenced by normalization of the following: (1) MITO biogenesis, (2) MITO fission and fusion, (3) MITO inner membrane integrity, and (4) CL synthesis and remodeling.

In the present study, dogs with HF demonstrated abnormalities of CL dynamics, specifically, decreased levels of total CL and (18:2)_4_CL, decreased CLS-1, and abnormalities in the CL remodeling enzymes TAZ-1 (decreased) and ALCAT-1 (increased). CL is found in high concentrations at contact sites between the inner and outer membranes of mitochondria, where fusion and fission occur [2]. CL plays a key role in MITO fusion via an interaction with OPA-1 and facilitates fission via recruitment and activation of Drp-1 [2]. Previous studies have shown that CL is decreased in diseases associated with MITO dysfunction and that the CL remodeling enzymes are either upregulated (ALCAT-1) or downregulated (TAZ-1) [2, 8]. The current study found that ELAM normalizes CL and (18:2)_4_CL, as well as the regulatory enzymes in dogs with HF compared with control animals with HF.

HF, regardless of etiology, is associated with increased sympathetic drive as evidenced by a sustained increase in PNE concentration. The increase in PNE can lead to downregulation of eNOS, decreased levels of cGMP, and finally to downregulation of PGC-1α, as illustrated in Fig. 1. Because PGC-1α is an important co-transcriptional regulator of MITO biogenesis, its downregulation can have a major adverse impact on MITO biogenesis and, therefore, lead to disruption of needed organelle turnover. Results from the present study indicate that long-term therapy with ELAM was associated with normalization of PNE concentration along with normalization of eNOS expression, cGMP levels, and expression of PGC-1α. Normalization of this signaling pathway affords a potential cardioprotective effect. In addition to its fundamental role in MITO biogenesis, PGC-1α is also an important regulator of lipid and glucose metabolism and data suggest that PGC-1α agonists can improve cardiac function, decrease fibrosis, and improve contractility and endothelial function in models of HF [25].

As alluded to earlier, results of the present study also demonstrated that MITO fission-regulating proteins, namely Fis-1 and Drp-1, are markedly increased whereas fusion-regulating proteins, namely Mfn2, OPA-1, are markedly decreased in dogs with HF and human hearts with DCM or ICM etiology. These findings are consistent with those reported in other animal models [1, 3, 26, 27]. Chen et al. [3] found that OPA-1 is decreased in HF in human and rat idiopathic cardiomyopathy and that the reduction was associated with increased apoptosis. Another group found that Mfn2/Drp-1 ratio (i.e., fusion/fission ratio) is decreased during HF and that treatment with a MITO division inhibitor improved cardiac function by normalizing the ratio [1]. The current study found that in dogs with HF, treatment with ELAM significantly increased Mfn2 and OPA-1 and significantly decreased Fis-1 and Drp-1 compared with HF-CON dogs. Phosphorylation of Mfn2 has also been shown to be an important mediator of mitophagy [28]. Phosphorylation of Mfn2 mediates the cytosolic ubiquitin ligase Parkin recruitment to damaged mitochondria [ 28]. Ablation of Mfn2 in mouse cardiomyocytes was shown to suppress mitophagy [28]. In the present study, pMfn2 was significantly downregulated, a condition that likely suppressed mitophagy and gave rise, in part, to the accumulation of morphologically and functionally abnormal mitochondria.

Our results also indicate that the inner membrane protein mitofilin was significantly reduced in HF-CON dogs compared with normal dogs and in human DCM and ICM hearts compared with DNR human hearts. Mitofilin is a protein of the inner MITO membrane and is associated with a large multimeric protein complex of about 1200 kDa. Mitofilin has critical functions in MITO morphology and MITO fission and fusion, specifically in the formation of tubular cristae and cristae junctions. Mitofilin also regulates cytochrome C release during apoptosis. Downregulation of mitofilin in HeLa cells has been shown to lead to decreased cellular proliferation and increased apoptosis, along with ultrastructural evidence of disorganized MITO inner membrane; abnormalities that are also manifested in HF. Mitofilin is one of the most abundant MITO proteins and is highly expressed in heart muscle [29]. Downregulation of mitofilin is invariably associated with MITO dysfunction. Mitofilin has been shown to be diminished in diabetic cardiomyopathy [30]. Results from our study demonstrate that administration of ELAM is associated with a normalization of mitofilin abundance in dogs with HF compared with untreated dogs with HF.

The present study has some limitations to be considered. We previously showed that the dog model of chronic HF from which LV tissue was obtained for the present study manifest abnormalities of cardiomyocyte MITO ultrastructure characterized by small mitochondria with loss of electron dense matrix and disrupted inner membrane [ 31]. We also showed that these ultrastructural abnormalities are associated with abnormal MITO function characterized by poor MITO respiration, reduced membrane potential and reduced maximum rate of ATP synthesis [22, 32]. In the present study, we did not obtain and specifically prepare tissue to evaluate MITO ultrastructure by transmission electron microscopy (TEM). Therefore, we were unable to demonstrate that normalization of fission and fusion proteins, mitofilin, and cardiolipin synthesis and remodeling proteins after treatment with ELAM also resulted in normalization of MITO ultrastructure. Nonetheless, several studies by other investigators have shown MITO ultrastructure normalization in multiple organs following therapy with ELAM [32–36].. In mice with HF produced by transverse aortic constriction (TAC), saline treated control animals showed increased number of damaged MITO with disrupted cristae compared to normal cristae in animals treated with SS-31 or ELAM [33].. Treatment with ELAM was also shown to normalize MITO morphology in kidneys of aging mice, in rats with acute kidney injury due to ischemia reperfusion, and improved MITO ultrastructure and morphology in lymphoblasts and fibroblasts derived from patients with Friedreich ataxia [34–37]**.**

Taken together, the results of the current study provide further evidence of the role of MITO abnormalities in the progression of the HF state and demonstrate that long-term therapy with ELAM reverses these abnormalities. The results provide additional mechanistic framework for the improvements in LV function associated with ELAM therapy in dogs with coronary microembolization-induced HF and suggest that future investigations of ELAM in the treatment of HF in humans are warranted.
